# Text message-based diabetes self-management support (SMS4BG): study protocol for a randomised controlled trial

**DOI:** 10.1186/s13063-016-1305-5

**Published:** 2016-04-02

**Authors:** Rosie Dobson, Robyn Whittaker, Yannan Jiang, Matthew Shepherd, Ralph Maddison, Karen Carter, Richard Cutfield, Catherine McNamara, Manish Khanolkar, Rinki Murphy

**Affiliations:** National Institute for Health Innovation, University of Auckland, Private Bag 92019, Auckland, 1142 New Zealand; Waitemata District Health Board, Auckland, New Zealand; School of Counselling, Human Services and Social Work, Faculty of Education, University of Auckland, Auckland, New Zealand; Auckland District Health Board, Auckland, New Zealand; School of Medicine, Faculty of Medical and Health Sciences, University of Auckland, Auckland, New Zealand

**Keywords:** mHealth, Diabetes mellitus, Text message, Mobile phone, SMS, Self-management

## Abstract

**Background:**

Addressing the increasing prevalence, and associated disease burden, of diabetes is a priority of health services internationally. Interventions to support patients to effectively self-manage their condition have the potential to reduce the risk of costly and debilitating complications. The utilisation of mobile phones to deliver self-management support allows for patient-centred care at the frequency and intensity that patients desire from outside the clinic environment. Self-Management Support for Blood Glucose (SMS4BG) is a novel text message-based intervention for supporting people with diabetes to improve self-management behaviours and achieve better glycaemic control and is tailored to individual patient preferences, demographics, clinical characteristics, and culture. This study aims to assess whether SMS4BG can improve glycaemic control in adults with poorly controlled diabetes. This paper outlines the rationale and methods of the trial.

**Methods/design:**

A two-arm, parallel, randomised controlled trial will be conducted across New Zealand health districts. One thousand participants will be randomised at a 1:1 ratio to receive SMS4BG, a theoretically based and individually tailored automated text message-based diabetes self-management support programme (intervention) in addition to usual care, or usual care alone (control). The primary outcome is change in glycaemic control (HbA1c) at 9 months. Secondary outcomes include glycaemic control at 3 and 6 months, self-efficacy, self-care behaviours, diabetes distress, health-related quality of life, perceived social support, and illness perceptions. Cost information and healthcare utilisation will also be collected as well as intervention satisfaction and interaction.

**Discussion:**

This study will provide information on the effectiveness of a text message-based self-management support tool for people with diabetes. If found to be effective it has the potential to provide individualised support to people with diabetes across New Zealand (and internationally), thus extending care outside the clinic environment.

**Trial registration:**

Australian New Zealand Clinical Trials Registry: ACTRN12614001232628.

**Electronic supplementary material:**

The online version of this article (doi:10.1186/s13063-016-1305-5) contains supplementary material, which is available to authorized users.

## Background

Addressing increasing diabetes prevalence, its associated morbidity and health inequalities, is a current priority in New Zealand and internationally [1–4]. The burden of diabetes is greater in indigenous peoples [1] with higher disease prevalence and poorer outcomes seen in Māori [5, 6]. Good diabetes self-management, including glucose monitoring, engaging in health behaviours, insulin administration, and healthcare provider contact, is associated with improved glycaemic control, and even small improvements in glycaemic control are associated with reduction in costly and debilitating long-term complications [7–12].

Mobile phones are ubiquitous, with increasing ownership and use across hard-to-reach populations [13, 14]. Text message (short message service, SMS) volumes have remained high over recent years with nearly 14 billion SMS messages sent in New Zealand in the year ending June 2012 [15]. Mobile health (mHealth) is the use of mobile devices, including mobile phones, to deliver health services and information [16]. Mobile phones have been used effectively to support healthy behaviour change and disease management [17–21], and offer an ideal way of providing patient-centred care at the frequency and intensity that patients desire. In addition there is potential for mobile phones to provide an effective way of providing support to patients in rural and remote areas where healthcare provider contact may be less accessible [22, 23].

There is a growing body of evidence supporting the use of mobile phones in the management of diabetes [24–26], with previous studies showing positive impacts on glycaemic control [25, 27], patient satisfaction with healthcare [25], healthcare costs [25], self-efficacy [28], and self-management behaviours, e.g. adherence and blood glucose monitoring [28–30]. Although promising, many previous studies have lacked sufficient sample sizes, were of insufficient duration, or interventions lacked theoretical grounding. Learnings has identify that for this type of intervention to be successful it needs to be theoretically based [19, 31], individually tailored [22, 32–35], and to provide individual choice to increase patients’ sense of control [36].

In light of the increasing prevalence of diabetes in New Zealand, as well as increasing mobile phone penetration, we hypothesise that mobile-based tools are important options for self-management support in this population. A mobile phone-based text messaging programme designed to enhance self-management support for people with diabetes (SMS4BG: Self-Management Support for Blood Glucose) has been developed and piloted [37]. Development of the SMS4BG followed the mHealth Development and Evaluation framework [38], with a focus on implementation, use of behavioural change theory, and engagement of key stakeholders including clinicians and patients. Conceptualisation, formative research and pretesting, including a pilot study, have been previously reported [37, 39].

SMS4BG was developed by a multidisciplinary team including public health and mHealth experts, psychologists, diabetes nurse specialists, a Māori advisory group, with review and input from diabetes specialists and primary care teams. The intervention is grounded in behaviour change theory to enhance people’s self-efficacy [40], and to promote accurate illness perceptions [41]. It uses Behaviour Change Techniques (BCTs) [42] (see Additional file 1 for a list of BCTs utilised in SMS4BG) to address the behaviours required for successful self-management and is made up of modules allowing for tailoring to the individual patient. The intervention content is designed to address the seven key self-management behaviours identified by the Association of American Diabetes Educations: (1) healthy eating, (2) being active, (3) monitoring, (4) taking medication, (5) problem solving, (6) reducing risks, and (7) healthy coping [43]. In addition the intervention includes versions for Māori and Pacific peoples incorporating concepts and elements specific to these cultures. Involvement of primary and secondary care teams throughout the development attempts to ensure integration into clinical pathways. The pilot study found the programme acceptable, useful, and culturally and age-appropriate [37]. Feedback from the SMS4BG pilot study allowed for further development and refinement of SMS4BG including increasing the duration of the programme based on patient preference, the addition of new modules including a foot care module and a cardiovascular check reminder module, and increased tailoring to incorporate individual motivations and names of support people. While the pilot study yielded positive results, a larger-scale randomised controlled trial (RCT) of the effectiveness of SMS4BG is now required including the effectiveness of SMS4BG in urban, and rural and remote areas. The findings from this RCT will inform the decision on whether to scale up and implement the programme across New Zealand.

### Aim

This study aims to determine the effectiveness of the mHealth diabetes self-management support programme (SMS4BG) in adults with poorly controlled type 1 or type 2 diabetes, in addition to their usual diabetes care. Specific objectives include:

1. Enabling improved diabetes self-management as measured by improvements in glycosylated haemoglobin (HbA1c)
2. Assessing the effectiveness of SMS4BG in areas with high rural/remote populations

## Methods/design

This protocol describes a 9-month, two-arm, parallel, RCT to evaluate the effectiveness of a text message-based diabetes self-management support programme (SMS4BG), on glycaemic control as measured by HbA1c. This protocol is in accord with the Standard Protocol Items: Recommendations for Interventional Trials (SPIRIT) 2013 statement [44], and the intervention is described according to the Consolidated Standards of Reporting Trials (CONSORT)-EHEALTH checklist [45]. See Additional file 2 for the completed SPIRIT checklist.

### Study population and recruitment

Eligible participants are adults (aged 16 years and over) with poorly controlled diabetes (defined as an HbA1c over 65 mmol/mol in the preceding 9 months) who own a text message-capable mobile phone, are able to read English, provide informed consent, and are available for the study duration. Exclusion criteria are not being available for the duration of the study, or being unable to use a mobile phone due to physical disabilities affecting eyesight or dexterity and not having a carer who wishes to use the mobile tools on their behalf. Recruitment for the trial commenced June 2015.

Potential participants will be identified by clinicians within primary and secondary care services across New Zealand health districts. Districts will be categorised as either high urban population or high rural/remote population based on population destiny data. Recruitment processes will build on those used successfully in the pilot study with clinicians forwarding the contact details of interested and eligible participants to the research team who will contact the patient by phone to discuss the study and gain informed consent. Informed consent will be obtained from all participants before they are enrolled in the study.

### Outcome assessments

**Fig. 1:**
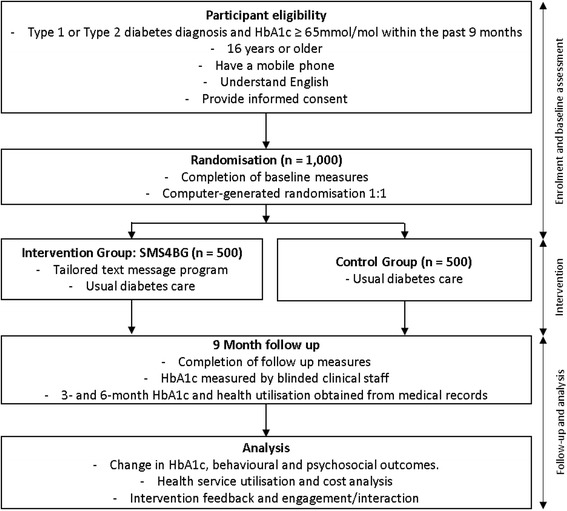
Study flow diagram

Assessments will be conducted at baseline and 9 months post randomisation (see Fig. 1). Baseline assessments will involve collection of demographic information and self-reported outcome measures via phone interview, and collection of clinical measures via patient records. Following completion of baseline data collection the patient will be randomised and, if allocated to the intervention arm, will be asked a small number of intervention-tailoring questions and will then be enrolled in the intervention. Intervention-tailoring questions include:

- Preferred first name
- Preferred mobile number
- Preferred message delivery time (early morning: 7–9 am, mid-morning: 9 am–12 pm, early afternoon: 12–3 pm, late afternoon: 3–5 pm, evening: 5–8 pm, or day: 9 am–5 pm)
- Region (Auckland or non-Auckland)
- Names and relationship of two support people (partner, parent/caregiver, child, friend, other)
- Motivations for diabetes management (your family, your *whānau*, your partner, your husband, your wife, your mum, your dad, your child, your children, your grandchild, your grandchildren, your friends, your career, your health in the future, sport, your fitness)
- Module choices (See Table 1).

**Table 1:** Description of SMS4BG modules

Module	Description	Participants	Duration	Example message
Core module	Two messages per week providing general motivation and support for diabetes management. Available in 3 version: Māori Pacific Non-Māori/non-Pacific	All	3 to 9 months	SMS4BG: *Kia ora*. Control of your glucose levels involves eating the right *kai*, exercise & taking your medication. Your *whānau*, doctor & nurse can help you
				SMS4BG: *Talofa* (name). by managing your diabetes well (including eating well and exercising) you can show your family that diabetes can be controlled
				SMS4BG: There is no quick fix to diabetes but with good management it will have less impact on your life and leave you more time to do the things you enjoy
Insulin module	One educational text message per week around insulin management and hypos for patients receiving insulin	Available to participants who are prescribed insulin	3 to 9 months	SMS4BG: Keep unopened insulin in the fridge. Don’t use insulin that has changed colour, is lumpy, expired, cracked or leaking, or has been frozen or overheated
Young adult module	One message per week around managing diabetes in the context or work/school and social situations	Available to participants aged 16–24	3 to 9 months	SMS4BG: Unsure whether to tell your friends/boyfriend/girlfriend about diabetes? This can be tough but people who care about you will want to know & support you
Smoking cessation module	One message per month encouraging participants to consider quitting smoking and providing details of services for support	All participants who register as smokers	3 to 9 months	SMS4BG: (hi) (name). Good management of your diabetes & your future health includes not smoking, call Quitline on 0800 778 778 for support
Lifestyle behaviour modules	Up to 4 messages per week encouraging participants to set a lifestyle goal and supporting them to work towards this goal. Participants can receive one of these modules for 3 months. The three lifestyle modules are: Healthy eating Exercise Stress and mood	Available to all participants	Each module 3 months	SMS4BG: Healthy eating is an important part of your diabetes treatment and it will help you in controlling your blood glucose levels
				SMS4BG: If you are too tired to exercise at the end of the day, try waking up early & doing your exercise in the morning. It will energise you for the day
				SMS4BG: (hi) (name). Make sure you have fun activities scheduled regularly. Doing something enjoyable helps reduce stress & improves mood
Blood glucose monitoring	Reminders to test blood glucose, sent at a frequency selected by the patient, for which they are encouraged to respond by reply text message with their blood sugar readings. In addition informational messages around managing hypos.	Available to all participants required to monitor their blood glucose	3 to 9 months	SMS4BG: (hi) (name). Just a reminder it is time to check your blood glucose. Reply with the result.
				If valid response received to reminder, ‘SMS4BG: Thank you for sending your result’
				SMS4BG: Hypoglycaemias (hypos) are when your blood glucose drops too low (i.e. less than 4 mmol/L). If this happens take something with sugar immediately
Foot care module	Reminders and motivational messages supporting engagement in foot care	Available to those who are classified as high risk or have active foot disease	3 to 9 months	SMS4BG: Looking after your feet will help to prevent issues in the future. Check your feet daily & contact your doctor, nurse or podiatrist if there are changes
Cardiovascular check reminder	Reminder to engage in a yearly cardiovascular assessment	Available to those who qualify	3 months	SMS4BG: (hi) (name). Next time you see your doctor ask about getting a cardiovascular check done. You should have one each year

Follow-up assessments will involve completion of self-reported outcome measures via phone interview, and collection of clinical measures via patient records.

### Ethics approval

Ethical approval for this trial was obtained from the Health and Disability Ethics Committee (14/STH/162).

### Sample size

One thousand participants (500 per arm) will be recruited for the trial, stratified by the health districts with either high urban or high rural/remote populations. Recruiting 500 participants (250 per arm) in each of the health district populations will provide 90 % power at the 5 % significance level to detect an overall clinically meaningful difference of 0.5 % (6 mmol/mol) change in HbA1c from baseline to 9 months between the two arms in each of the populations, assuming a standard deviation of 1.7 %. Targeted recruitment strategies will be used to preferentially recruit Māori and Pacific participants where possible.

### Randomisation and blinding

Eligible participants will be randomised to either intervention or control group in a 1:1 ratio. Randomisation will be stratified by health district category (high urban or high rural/remote), diabetes type (1 or 2), and ethnicity (Māori and Pacific, or non-Māori and non-Pacific). The randomisation sequence will be generated by computer programme using variable block sizes of 2 or 4, and overseen by the study statistician (YJ). The treatment allocation will be concealed until the point of randomisation. Due to the nature of the intervention participants will be aware of their treatment allocation. Although it will not be possible for research staff conducting the phone interviews to be blinded to the treatment allocation, the primary outcome HbA1c is an objective measure and assessors of this outcome will be blinded to treatment allocation.

### Intervention

Both intervention and control groups will continue with their usual diabetes care including all medical visits, tests, and diabetes support programmes. In addition the intervention group will receive the automated text message-based self-management support programme (SMS4BG) for up to 9 months. SMS4BG is tailored according to the needs and goals of the patient, their care plan, and demographic factors including ethnicity. As well as core motivational and support messages (available in Māori, Pacific and non-Māori/non-Pacific versions), participants can opt to receive additional modules such as a lifestyle module around healthy eating, physical activity or stress management. Where appropriate to their care, they can also receive reminders to check blood glucose levels, messages around insulin, foot care, managing diabetes as a young adult, and smoking cessation, and also messages encouraging preventive behaviours (e.g. cardiovascular risk assessment). Participants who opt to reply to glucose monitoring reminders, by sending in their blood glucose levels by text message, will be able to view their blood glucose levels graphically over time on a secure website. If at baseline they are identified as not having access to the Internet they will be mailed their graphs on a monthly basis. At registration the intervention group will be able to select the timing of their messages and blood glucose monitoring reminders, and to identify their support people and motivations for diabetes management for incorporation into the messages. The length of the programme will also be tailored to patient preferences from 3 to 9 months, and at 3 and 6 months participants will receive a text message asking if they would like to continue the programme for an additional 3 months and will be given the opportunity to re-select their modules. Participants can stop their messages at any time by texting the word ‘STOP’, or put their messages on hold for 1 week by sending the text word ‘HOLIDAY’. A summary of the structure of SMS4BG can be seen in the Table 1. More detail on the intervention and its development is available in the pilot study paper [37].

The message delivery will be managed by a specifically developed SMS4BG Content Management System with the messages sent and received through a gateway company to allow for participants to be registered with any New Zealand mobile network. Sending and receiving messages will be free to all participants with costs covered by the study. The system will maintain logs of all outgoing and incoming messages, and incoming blood glucose values will be automatically graphed by the system which individuals can view via a password-protected website.

### Outcome measures

The primary outcome measure is change in glycaemic control from baseline to 9 months, measured as HbA1c (in mmol/mol or %) by registered laboratories. Secondary outcome measures include:

- *Glycaemic control* measured by registered laboratory measurements of HbA1c (in mmol/mol or %) at 3 months and at 6 months. Both 3-month and 6-month HbA1c results will be obtained from patient records at the 9-month follow-up
- *Self-efficacy for diabetes management* measured by the Stanford Self-efficacy for Diabetes Management scale (SEDM) [46] at baseline and at 9 months. The SEDM is an 8-item measure which respondents use to indicate, on a Likert scale from 1 (not at all confident) to 10 (totally confident), how confident they feel that they can carry out the listed tasks regularly at the present time. The score is calculated using the mean of the eight items with higher scores indicating higher self-efficacy. The SEDM has been found to have good internal consistency and test-retest validity [46]
- *Diabetes self-care behaviours* measured by the Summary of Diabetes Self-Care Activities [47] (SDSCA) at baseline and at 9-month follow-up. The SDSCA is a brief self-reported questionnaire which asks respondents 11 items relating to five different domains of diabetes self-management: diet, exercise, blood-glucose monitoring, foot care, and smoking. The SDSCA has been shown to have good validity and reliability in research and practice [47], with higher scores on the scale indicating greater engagement in self-care behaviours
- The presence of *diabetes-related distress* measured by the 2-item Diabetes Distress Scale (DDS2) [48] at baseline and at 9 months. This 2-item brief diabetes distress screening instrument detects diabetes-specific distress. Respondents indicate, on a 6-point Likert scale, to what degree each item has caused them distress over the past month with higher score indicating higher distress. The DDS2 has been shown to discriminate highly distressed patients from patients with low diabetes distress [48], with an average item score of 3 or more used as the cut off for high distress
- *Cognitive and emotional representations of diabetes* measured by the Brief Illness Perception Questionnaire (BIPQ) [49] at baseline and at 9 months. The BIPQ is a 9-item self-reported measure which assesses consequences, timeline, personal control, treatment control, identity, concern, emotions, illness comprehensibility, and causes of diabetes. Each item (except causality) is rated using an 11-point Likert scale with higher scores indicating greater agreement with the item. The causal representation is assessed via an open-ended item. The BIPQ has been shown to have good reliability and validity [49], and has previously been used in a New Zealand diabetes population to assess differences in illness perceptions between Europeans, South Asians and Pacific Islanders [50]
- *Health-related quality of life* measured by the EuroQol 5 dimensions (EQ-5D) questionnaire [51] at baseline and at 9-month follow-up. The EQ-5D provides a descriptive profile of health status and a single index value for health status. Five dimensions of health are assessed in the descriptive system: mobility, self-care, usual activities, pain/discomfort and anxiety/depression. The respondent indicates under each dimension their health state by choosing the severity level most appropriate to themselves. A lower number indicates a better health status and quality of life. The EQ-5D visual analogue scale allows the respondent to mark, on a scale from 0 (worst imaginable health state) to 100 (best imaginable health state), their health state. There is evidence to support the validity and reliability of the EQ-5D in people with diabetes [52]
- *Perceived social support for diabetes management* measured using a 4-item measure developed for this study at baseline and at 9-month follow-up. The measure is split into two sections. The first assesses general support and asks how supported they feel in regards to their diabetes management on a 6-point Likert scale, from 1 (not at all supported) to 6 (extremely supported). The second section assesses appraisal, emotional and advice/information aspects of support (one item each). Users indicate, on a 6-point Likert scale ranging from 1 (strongly disagree) to 6 (strongly agree), to what degree they agree with the statements
- *Healthcare utilisation* measured by number of hospitalisations, and primary and secondary care visits during the study period compared to the 9 months prior to randomisation. Healthcare utilisation will be obtained from patient records at 9-month follow-up
- *Patient satisfaction and engagement* with SMS4BG (for those in the intervention group). At 9 months participants will be asked, via semi-structured interview, about their satisfaction with the programme, including ease of use, issues arising, satisfaction with the text messages, salience and usefulness of the messages, and suggestions for improvement. In addition the overall number of text messages sent and received, response rates, and intervention duration will be measured by the SMS4BG content management system
- *Cost-effectiveness* of the intervention using cost information obtained at 9 month follow-up, including the costs of the SMS4BG programme, the direct medical costs (including cost of treatment, primary care, secondary care) and Quality-adjusted Life Years (QALYs)

### Statistical analysis

Statistical analyses will be performed using SAS version 9.4 (SAS Institute Inc. Cary, NC, USA). All statistical tests will be two-sided at the 5 % significance level. All treatment evaluations will be performed on the principle of intention-to-treat (ITT), using the observed data collected from all randomised participants. Appropriate imputation methods will be applied to the missing data on the primary outcome. No imputation will be considered on other secondary outcomes. A per-protocol analysis may be conducted on the subset of participants who are more compliant with the protocol with pre-defined criteria.

Demographics and baseline characteristics will be summarised using descriptive statistics. Continuous variables will be summarised as numbers of observed values, mean, standard deviation, median, minimum and maximum. Categorical variables will be described as frequency and percentage. Information collected on all primary and secondary outcomes will be first summarised using descriptive statistics at baseline and at 9 months as appropriate. Results will be presented for each of the two treatment groups separately. Linear regression model will be used to test the effect of intervention on the primary outcome between two groups, adjusting for baseline outcome value, health district category, type of diabetes, and ethnicity (i.e. the stratification factors). Model-adjusted means and their difference will be presented with 95 % confidence intervals. As pre-planned, the analysis will also be conducted for each health district category separately as stratified, and the consistency of intervention effects will be tested in the main model using an interaction term between treatment group and health district category. A similar approach will be applied to other continuous secondary outcomes. Generalised linear regression models will be applied to categorical outcomes using an appropriate link function (e.g. a logit link for binary distribution).

Sensitivity analysis may be conducted on the primary outcome if the proportion of missing data is greater than 10 %. Both single and multiple imputations’ methods may be considered based on different assumptions on the missing data, in order to assess the robustness of treatment evaluation.

If enough participants are recruited, subgroup analyses by diabetes type and ethnic group will be conducted on the primary outcome and key secondary outcomes, to test possible interactions with the intervention.

## Discussion

This paper describes the protocol for the SMS4BG trial to evaluate a text message-based diabetes self-management support programme compared with usual care. This type of intervention can provide tailored support between clinic visits for people with poorly controlled diabetes. This protocol builds on previous evidence for the role of mHealth in people with diabetes. The SMS4BG study has been designed to address limitations of previous diabetes text message studies. The intervention, developed by a multidisciplinary team, is comprehensive in design, individually tailored, and theoretically grounded, and the study design will allow for accurate assessment of the impact of this type of intention.

Findings from the pilot study of SMS4BG show that this type of intervention is acceptable and perceived as useful by people with diabetes in New Zealand although the effectiveness must be proven in a rigorously conducted trial. If found to be effective, SMS4BG has the potential to be implemented into health services across New Zealand and the potential to be adapted for other populations.

### Trial status

Recruiting: participants are currently being recruited and enrolled.

## Additional files

Additional file 1: List of Behaviour Change Techniques (BCT) utilised in SMS4BG by grouping (BCT Taxonomy v1). (PDF 67 kb) Additional file 2: SPIRIT 2013 Checklist. Completed SPIRIT checklist. (PDF 82 kb)

## Abbreviations

BCT: Behaviour Change Technique
BIPQ: Brief Illness Perception Questionnaire
DDS2: Diabetes Distress Scale – 2-item
DHB: District Health Board
ITT: intention-to-treat
QALY: Quality-adjusted Life Year
RCT: randomised controlled trial
SDSC: Summary of Diabetes Self-Care Activities
SEDM: Self-efficacy in Diabetes Management scale
SMS: short messaging service
SMS4BG: self-management support for blood glucose

## References

[1] International Diabetes Federation. IDF Diabetes Atlas, 6th ed. Brussels, Belgium. http://www.diabetesatlas.org/: International Diabetes Federation 2013. Accessed 17 Nov 2015.

[2] Ministry of Health. Virtual Diabetes Register (VDR): Estimated diagnosed cases of diabetes by DHB as at December 2013. 2013. http://www.health.govt.nz/our-work/diseases-and-conditions/diabetes/about-diabetes/virtual-diabetes-register-vdr/2013-virtual-diabetes-register-results. Accessed 17 Nov 2015.

[3] Coppell KJ, Mann JI, Williams SM, Jo E, Drury PL, Miller JC (2013). Prevalence of diagnosed and undiagnosed diabetes and prediabetes in New Zealand: findings from the 2008/09 Adult Nutrition Survey. New Zeal Med J.

[4] Joshy G, Simmons D (2006). Epidemiology of diabetes in New Zealand: revisit to a changing landscape. New Zeal Med J.

[5] Ministry of Health (2013). New Zealand Health Survey: annual update of key findings 2012/13.

[6] Jeffreys M, Wright C, Huang K, Pearce N (2005). Ethnic differences in cause specific mortality among hospitalised patients with diabetes: a linkage study in New Zealand. J Epidemiol Community Health.

[7] Morrison F, Shubina M, Turchin A (2011). Encounter frequency and serum glucose level, blood pressure, and cholesterol level control in patients with diabetes mellitus. Arch Intern Med.

[8] Karter AJ, Ackerson LM, Darbinian JA, D’Agostino RB, Ferrara A, Liu J (2001). Self-monitoring of blood glucose levels and glycemic control: the Northern California Kaiser Permanente Diabetes Registry. Am J Med.

[9] Bailey TS, Zisser HC, Garg SK (2007). Reduction in hemoglobin A1C with real-time continuous glucose monitoring: results from a 12-week observational study. Diabetes Technol Ther.

[10] Cho J-H, Chang S-A, Kwon H-S, Choi Y-H, Ko S-H, Moon S-D (2006). Long-term effect of the Internet-based glucose monitoring system on HbA1c reduction and glucose stability. A 30-month follow-up study for diabetes management with a ubiquitous medical care system. Diabetes Care.

[11] Stratton IM, Adler AI, Neil HAW, Matthews DR, Manley SE, Cull CA (2000). Association of glycaemia with macrovascular and microvascular complications of type 2 diabetes (UKPDS 35): prospective observational study. BMJ.

[12] The Diabetes Control and Complications Trial Research Group (1993). The effect of intensive treatment of diabetes on the development and progression of long-term complications in insulin-dependent diabetes mellitus. N Engl J Med.

[13] Statistics New Zealand (2010). Household use of information and communication technology: 2009.

[14] Research New Zealand. The rise and rise of smartphones and other mobile devices: Media Release; 5 March 2013. URL: http://www.researchnz.com/pdf/Media%20Releases/RNZ%20Media%20Release%20-%20Penetration%20and%20use%20of%20electronic%20devices.pdf. [Accessed 26 Aug 2014] [WebCite Cache ID 6S6jiLwcI].

[15] Commerce Commission New Zealand. Annual Telecommunications Monitoring Report 2012. Wellington, New Zealand: Commerce Commision New Zealand; 2013 Apr. URL: www.comcom.govt.nz/dmsdocument/10043. [Accessed 24 Feb 2015] [WebCite Cache ID 6WakI9kF0].

[16] Global Observatory for eHealth Series - Volume 3. Geneva, Switzerland: WHO Press; 2011. mHealth: New horizons for health through mobile technologies URL: http://www.who.int/goe/publications/goe_mhealth_web.pdf. [Accessed 14 Jan 2015] [WebCite Cache ID 6VaF4Zrjl].

[17] Cole-Lewis H, Kershaw T (2010). Text messaging as a tool for behavior change in disease prevention and management. Epidemiol Rev.

[18] Hall AK, Cole-Lewis H, Bernhardt JM (2015). Mobile text messaging for health: a systematic review of reviews. Annu Rev Public Health.

[19] Free C, Phillips G, Galli L, Watson L, Felix L, Edwards P (2013). The effectiveness of mobile-health technology-based health behaviour change or disease management interventions for health care consumers: a systematic review. PLoS Med.

[20] Whittaker R, McRobbie H, Bullen C, Borland R, Rodgers A, Gu Y. Mobile phone‐based interventions for smoking cessation. Cochrane Database Syst. Rev. 2012;11. doi:10.1002/14651858.CD006611.pub3 10.1002/14651858.CD006611.pub3 23152238

[21] Krishna S, Boren SA, Balas EA (2009). Healthcare via cell phones: a systematic review. Telemed J E Health.

[22] Heron KE, Smyth JM (2010). Ecological momentary interventions: incorporating mobile technology into psychosocial and health behaviour treatments. Br J Health Psychol.

[23] Boland P (2007). The emerging role of cell phone technology in ambulatory care. J Ambul Care Manage.

[24] Liang X, Wang Q, Yang X, Cao J, Chen J, Mo X (2011). Effect of mobile phone intervention for diabetes on glycaemic control: a meta-analysis. Diabet Med.

[25] Nundy S, Dick JJ, Chou C-H, Nocon RS, Chin MH, Peek ME (2014). Mobile phone diabetes project led to improved glycemic control and net savings for Chicago plan participants. Health Aff.

[26] Russell-Minda E, Jutai J, Speechley M, Bradley K, Chudyk A, Petrella R (2009). Health technologies for monitoring and managing diabetes: a systematic review. J Diabetes Sci Technol.

[27] Rami B, Popow C, Horn W, Waldhoer T, Schober E (2006). Telemedical support to improve glycemic control in adolescents with type 1 diabetes mellitus. Eur J Pediatr.

[28] Franklin VL, Waller A, Pagliari C, Greene SA (2006). A randomized controlled trial of Sweet Talk, a text‐messaging system to support young people with diabetes. Diabet Med.

[29] Arora S, Peters AL, Burner E, Lam CN, Menchine M (2014). Trial to examine text message-based mHealth in emergency department patients with diabetes (TExT-MED): a randomized controlled trial. Ann Emerg Med.

[30] Hanauer DA, Wentzell K, Laffel N, Laffel LM (2009). Computerized Automated Reminder Diabetes System (CARDS): e-mail and SMS cell phone text messaging reminders to support diabetes management. Diabetes Technol Ther.

[31] Riley W, Rivera D, Atienza A, Nilsen W, Allison S, Mermelstein R (2011). Health behavior models in the age of mobile interventions: are our theories up to the task?. Transl Behav Med.

[32] Fjeldsoe BS, Marshall AL, Miller YD (2009). Behavior change interventions delivered by mobile telephone short-message service. Am J Prev Med.

[33] Noar SM, Benac CN, Harris MS (2007). Does tailoring matter? Meta-analytic review of tailored print health behavior change interventions. Psychol Bull.

[34] Ryan P, Lauver DR (2002). The efficacy of tailored interventions. J Nurs Scholarsh.

[35] Head KJ, Noar SM, Iannarino NT, Grant HN (2013). Efficacy of text messaging-based interventions for health promotion: a meta-analysis. Soc Sci Med.

[36] Mulvaney SA, Ritterband LM, Bosslet L (2011). Mobile intervention design in diabetes: review and recommendations. Curr Diab Rep.

[37] Dobson R, Carter K, Cutfield R, Hulme A, Hulme R, McNamara C, et al. Diabetes Text-Message Self-Management Support Program (SMS4BG): a pilot study. JMIR mHealth uHealth. 2015;3(1). doi:10.2196/mhealth.3988. 10.2196/mhealth.3988 PMC4390615 25830952

[38] Whittaker R, Merry S, Dorey E, Maddison R (2012). A development and evaluation process for mHealth interventions: Examples from New Zealand. J Health Commun.

[39] Whittaker R, Bramley D, Carter K, Cutfield R, Dobson R, Dorey E (2013). A comprehensive approach to self-management support for diabetic patients and clinicians..

[40] Bandura A (1989). Human agency in social cognitive theory. Am Psychol.

[41] Leventhal H, Brissette I, Leventhal EA (2003). The common-sense model of self-regulation of health and illness. The self-regulation of health and illness behaviour.

[42] Michie S, Richardson M, Johnston M, Abraham C, Francis J, Hardeman W (2013). The Behavior Change Technique Taxonomy (v1) of 93 hierarchically clustered techniques: building an international consensus for the reporting of behavior change interventions. Ann Behav Med.

[43] Funnell MM, Brown TL, Childs BP, Haas LB, Hosey GM, Jensen B (2009). National standards for diabetes self-management education. Diabetes Care.

[44] Chan A-W, Tetzlaff JM, Altman DG, Laupacis A, Gøtzsche PC, Krleža-Jerić K (2013). SPIRIT 2013 Statement: defining standard protocol items for clinical trials. Ann Intern Med.

[45] Eysenbach G, CONSORT-EHEALTH Group. CONSORT-EHEALTH: improving and standardizing evaluation reports of web-based and mobile health interventions. J Med Internet Res. 2011;13(4):e126. doi:10.2196/jmir.1923. 10.2196/jmir.1923 PMC3278112 22209829

[46] Lorig K, Ritter PL, Villa FJ, Armas J (2009). Community-based peer-led diabetes self-management: a randomized trial. Diabetes Educ.

[47] Toobert DJ, Hampson SE, Glasgow RE (2000). The summary of diabetes self-care activities measure: results from 7 studies and a revised scale. Diabetes Care.

[48] Fisher L, Glasgow RE, Mullan JT, Skaff MM, Polonsky WH (2008). Development of a brief diabetes distress screening instrument. Ann Fam Med.

[49] Broadbent E, Petrie KJ, Main J, Weinman J (2006). The brief illness perception questionnaire. J Psychosom Res.

[50] Bean D, Cundy T, Petrie KJ (2007). Ethnic differences in illness perceptions, self-efficacy and diabetes self-care. Psychol Health.

[51] The EuroQol Group (1990). EuroQol – a new facility for the measurement of health-related quality of life. Health Policy.

[52] Janssen M, Lubetkin E, Sekhobo J, Pickard A (2011). The use of the EQ‐5D preference‐based health status measure in adults with type 2 diabetes mellitus. Diabet Med.

