# Supplementary material for: Text message-based diabetes self-management support (SMS4BG): study protocol for a randomised controlled trial
Source: Trials. 2016 Apr 2;17:179. doi: 10.1186/s13063-016-1305-5 (PMC4818933; doi:10.1186/s13063-016-1305-5)
Supplement: Additional file 1: — List of Behaviour Change Techniques (BCT) utilised in SMS4BG by grouping (BCT Taxonomy v1). (PDF 67 kb) [file 13063_2016_1305_MOESM1_ESM.pdf]

Additional file 1: List of Behaviour Change Techniques utilized in SMS4BG by grouping (BCT Taxonomy v1) [42]

BCT Grouping	BCT
**1. Goals and planning**	1.1. Goal setting (behavior)
	1.2. Problem solving
	1.3. Action planning
**2. Feedback and monitoring**	2.3. Self-monitoring of behaviour
	2.4. Self-monitoring of outcome(s) of behaviour
**3. Social support**	3.1. Social support (unspecified)
	3.2. Social support (practical)
	3.3. Social support (emotional)
**4. Shaping knowledge**	4.1. Instruction on how to perform the behaviour
	4.2. Information about Antecedents
**5. Natural consequences**	5.1. Information about health consequences
	5.2. Salience of consequences
	5.3. Information about social and environmental consequences
	5.6. Information about emotional consequences
**7. Associations**	7.1. Prompts/cues
**8. Repetition and substitution**	8.2. Behaviour substitution
	8.4. Habit reversal
	8.7 Graded tasks
**11. Regulation**	11.2. Reduce negative emotions
	11.3. Conserving mental resources
**12. Antecedents**	12.1. Restructuring the physical environment
	12.2. Restructuring the social environment
	12.4. Distraction
**13. Identity**	13.1. Identification of self as role model
	13.2. Framing/reframing
**15. Self-belief**	15.1. Verbal persuasion about capability
