# Supplementary material for: Text message-based diabetes self-management support (SMS4BG): study protocol for a randomised controlled trial
Source: Trials. 2016 Apr 2;17:179. doi: 10.1186/s13063-016-1305-5 (PMC4818933; doi:10.1186/s13063-016-1305-5)
Supplement: Additional file 2: — SPIRIT 2013 Checklist. Completed SPIRIT checklist. (PDF 82 kb) [file 13063_2016_1305_MOESM2_ESM.pdf]

**Additional file 2: SPIRIT 2013 Checklist**

| Section/item | Item No | Included in manuscript (Y/N) or described below |
|---|---|---|
| **Administrative information** | | |
| Title | 1 | Y |
| Trial registration | 2a | Y |
| | 2b | Y |
| Protocol version | 3 | Version 4: 16/09/2015 |
| Funding | 4 | Y |
| Roles and responsibilities | 5a | Y |
| | 5b | Y |
| | 5c | Y |
| | 5d | The trial steering committee is comprised of the protocol authors. The National Institute for Health Innovation IT team is responsible for the SMS content management system and data management is overseen by the National Institute for Health Innovation data management team. |
| **Introduction** | | |
| Background and rationale | 6a | Y |
| | 6b | Y |
| Objectives | 7 | Y |
| Trial design | 8 | Y |
| **Methods: Participants, interventions, and outcomes** | | |
| Study setting | 9 | Y |
| Eligibility criteria | 10 | Y |
| Interventions | 11a | Y |
| | 11b | Y |
| | 11c | Y |
| | 11d | Y |
| Outcomes | 12 | Y |
| Participant timeline | 13 | Y |
| Sample size | 14 | Y |
| Recruitment | 15 | Y |
| **Methods: Assignment of interventions (for controlled trials)** | | |
| Allocation: | | |
| Sequence generation | 16a | Y |
| Allocation concealment mechanism | 16b | Y |
| Implementation | 16c | Y |
| Blinding (masking) | 17a | Y |
| | 17b | Y |
| Data collection methods | 18a | Y – All data collection forms are located in the Trial Master File |
| | 18b | Y |

| | | |
|---|---|---|
| Data management | 19 | Y – All data collection methods are described in the Manual of Procedures located in the trial master file. All data will be entered into REDCap. At least 10% of data in the electronic system will be checked and validated against the source document (where a hard copy exists). |
| Statistical methods | 20a | Y – The statistical methods are described in full in the Statistical Analysis Plan located in the Trial Master File |
| | 20b | Y |
| | 20c | Y |
| **Methods: Monitoring** | | |
| Data monitoring | 21a | The study does not meet two or more of the criteria outlined by Ellenburg et al. (2002) and therefore a DMC will not be established for the trial. An independent project monitor will be appointed. The monitor will be trained in GCP. The project is considered to be low risk. The monitor will check primary outcome measures, and TMF and consent forms for completeness and accuracy. |
| | 21b | NA – The trial is low risk and no interim analyses are planned |
| Harms | 22 | Y – The trial is low risk |
| Auditing | 23 | Y - The trial will be subject to independent auditing. |
| **Ethics and dissemination** | | |
| Research ethics approval | 24 | Y |
| Protocol amendments | 25 | Amendments will be communicated to the Trial Steering Committee, ethics boards and trial registry where needed. |
| Consent or assent | 26a | Y |
| | 26b | NA |
| Confidentiality | 27 | Y – Confidentiality will be protected by the use of study registration numbers, and only aggregated and anonymous data will be reported. Personal information will be kept confidential and stored securely. Computerised information will be password protected. All reports from the study will be written in a way that no individuals can be identified. Information about study subjects will be kept confidential in keeping with the obligations set out in the Privacy Act 1993, the Health Information Code 1994 and Section 22B to 221 |

| | | |
|---|---|---|
| | | of the Health Act 1956. Data will be entered, stored and backed-up in a secure manner on a server at the National Institute for Health Innovation. |
| Declaration of interests | 28 | Y |
| Access to data | 29 | Y |
| Ancillary and post-trial care | 30 | Described in the Participant Information Sheet and Consent Form located in the Trial Master File |
| Dissemination policy | 31a | Y – A copy of the trial Publication Plan included in the Trial Master File |
| | 31b | Outlined in the trial Publication Plan included in the Trial Master File. No professional writers will be used. |
| | 31c | Y – There is no plan to make the data set public |
| **Appendices** | | |
| Informed consent materials | 32 | Version 3, (27/02/2015) located in the Trial Master File |
| Biological specimens | 33 | NA |
